# Constraint-based causal discovery with mixed data

**DOI:** 10.1007/s41060-018-0097-y

**Published:** 2018-02-02

**Authors:** Michail Tsagris, Giorgos Borboudakis, Vincenzo Lagani, Ioannis Tsamardinos

**Affiliations:** 1 Department of Computer Science, University of Crete, Heraklion, Greece; 2 Gnosis Data Analysis (Gnosis DA), University of Crete, Heraklion, Greece; 3 Institute of Applied and Computational Mathematics, Foundation for Research and Technology, Hellas, Greece; 4 Huddersfield University, Yorkshire, UK

**Keywords:** Constraint-based learning, Bayesian networks, Maximal ancestral graphs, Mixed data, Conditional independence tests

## Abstract

We address the problem of constraint-based causal discovery with mixed data types, such as (but not limited to) continuous, binary, multinomial, and ordinal variables. We use likelihood-ratio tests based on appropriate regression models and show how to derive symmetric conditional independence tests. Such tests can then be directly used by existing constraint-based methods with mixed data, such as the PC and FCI algorithms for learning Bayesian networks and maximal ancestral graphs, respectively. In experiments on simulated Bayesian networks, we employ the PC algorithm with different conditional independence tests for mixed data and show that the proposed approach outperforms alternatives in terms of learning accuracy.

## Introduction

Typically, datasets contain different variable types, such as continuous (e.g., temperature), nominal (e.g., sex), ordinal (e.g., movie ratings), or censored time-to-event (e.g., customer churn), to name a few. Furthermore, data may be measured over time (e.g., longitudinal data) or without considering time (e.g., cross-sectional data). Such heterogeneous data are not exceptions, but the norm in many domains (e.g., biomedicine, psychology, and business). In such cases, it is important and necessary to apply causal discovery methods that are able to handle mixed data types.

Unfortunately, most current approaches do not handle heterogeneous variable types. Constraint-based methods, like the PC and FCI algorithms [37] for Bayesian network (BN) and maximal ancestral graph (MAG) learning, respectively, are general methods that use conditional independence tests to learn the causal network. Thus, in principle, they can be applied to heterogeneous variable types, as long as an appropriate conditional independence test is employed. For continuous variables, typical choices are the partial correlation test [3] or kernel-based tests [46]. Categorical variables are usually handled with the $X^2$ test or the *G* test [1]. Similarly, most score-based methods, such as the K2 [9] and GES [7] algorithms for BN learning, employ scores for categorical variables [9, 16] or for continuous variables only [14]. Although there exist both constraint-based [4, 10, 27] and score-based [4, 13, 15, 30] approaches for learning with mixed data, they are limited in the variable types they can handle and are too computationally expensive or make unrealistic assumptions.

In this work, we propose a simple and general method to handle mixed variables. We show how to deal with mixtures of continuous, binary, nominal, and ordinal variables, although the same methodology can be used to derive tests for other data types, such as count data, proportions (percentages), positive and strictly positive data, censored data, as well as robust versions for heteroscedastic data; see the R package MXM [24] for a list of available tests. Those tests can be directly plugged-in to existing constraint-based learning algorithms, such as the PC and FCI algorithms. Naturally, the proposed method is not limited to BN and MAG learning algorithms, but can be used with any algorithm that employs conditional independence tests, such as algorithms for Markov network structure discovery [6] or for feature selection [38].

We employ likelihood-ratio tests based on regression models to devise conditional independence tests for mixed data. A likelihood-ratio test for conditional independence of variables *X* and *Y* given a (possibly empty) set of variables $\mathbf {Z}$ can be performed by fitting two regression models for *X*, one using $\mathbf {Z}$ and one with $Y \cup \mathbf {Z}$, and comparing their goodness-of-fit. Under the null hypothesis of conditional independence, both models should fit the data equally well, as the inclusion of *Y* does not provide any additional information for *X* once $\mathbf {Z}$ is accounted for. Alternatively, one can flip *X* and *Y* and fit two regression models for *Y* instead. Unfortunately, those tests do not necessarily give the same results, especially for low sample scenarios, and thus are not symmetric. Symmetry is an important property, as the test decisions should not depend on the variable order.

In simulated experiments, we demonstrate that in the sample limit and by using appropriate regression models, both tests return the same *p* value and thus are asymptotically symmetric. To handle finite sample cases, we consider different approaches to obtain symmetry, such as performing both tests and combining them appropriately, or by performing only one test in an order-invariant fashion using predefined rules (similar to [34]). Finally, we evaluate two proposed symmetric tests (one of each category) against an alternative conditional independence test for mixtures of ordinal and continuous variables [10] on simulated BNs and show that the symmetric test based on performing two asymmetric likelihood-ratio tests, called MM, outperforms the rest.

## Preliminaries

### Bayesian networks and maximal ancestral graphs

A Bayesian network (BN) [31, 37] $B = \langle G, P \rangle$ consists of a directed acyclic graph *G* over vertices (variables) $\mathbf {V}$ and a joint probability distribution *P*. *P* is linked to *G* through the Markov condition, which states that each variable is conditionally independent of its nondescendants given its parents. The joint distribution *P* can then be written as

$$\begin{aligned} P(V_1, \dots , V_n) = \prod _{i=1}^p P\left( V_i | Pa(V_i)\right) , \end{aligned}$$

where *p* is the total number of variables in *G* and Pa($V_i$) denotes the parent set of $V_i$ in *G*. If all conditional independencies in *P* are entailed by the Markov condition, the BN is called faithful. Furthermore, BNs assume causal sufficiency, that is, that there are no latent confounders between variables in $\mathbf {V}$.

A causal BN is a BN where edges are interpreted causally. Specifically, an edge $X \rightarrow Y$ exists if *X* is a direct cause of *Y* in the context of the measured variables $\mathbf {V}$. Typically, multiple BNs encode the same set of conditional independencies. Such BNs are called Markov equivalent, and the set of all Markov equivalent BNs forms a Markov equivalence class. This class can be represented by a completed partially directed acyclic graph (PDAG), which in addition to directed edges also contains undirected edges. Undirected edges may be oriented either way in some BN in the Markov equivalence class (although not all combinations are possible), while directed and missing edges are shared among all equivalent networks.

Two classes of algorithms for BN learning are constraint-based and score-based methods. Constraint-based learning algorithms, such as the PC algorithm [37], employ conditional independence tests to discover the structure of the network, and perform an orientation phase afterward to orient (some of) the edges, and a PDAG is returned. Score-based methods [7, 9, 16] assign a score on the whole network based on how well it fits the data and perform a search in the space of BNs or PDAGs to identify a high-scoring network.

Maximal ancestral graphs (MAG) [33] are generalizations of BNs that admit the presence of latent confounders, and thus drop the causal sufficiency assumption. In addition to directed edges, they also contain bidirected edges, which encode dependencies due to latent confounders. As for BNs, multiple Markov equivalent networks may exist, forming a Markov equivalence class, which can be represented by a graph called partial ancestral graph (PAG). The FCI algorithm [37, 45], an extension of the PC algorithm, outputs such a PAG.

### Conditional independence tests

Let *X* and *Y* be two random variables, and $\mathbf {Z}$ be a (possibly empty) set of random variables. *X* and *Y* are conditionally independent given $\mathbf {Z}$, if $P(X,Y|\mathbf {Z}) = P(X|\mathbf {Z}) \cdot P(Y|\mathbf {Z})$ holds for all values of *X*, *Y*, and $\mathbf {Z}$. Equivalently, conditional independence of *X* and *Y* given $\mathbf {Z}$ implies $P(X|Y,\mathbf {Z}) = P(X|\mathbf {Z})$ and $P(Y|X,\mathbf {Z}) = P(Y|\mathbf {Z})$. Such statements can be tested using conditional independence tests. Examples of commonly employed conditional independence tests are the partial correlation test [3] for continuous multivariate Gaussian variables, and the *G* test and the (asymptotically equivalent) $X^2$ test [1, 37] for categorical variables. All aforementioned tests are either likelihood-ratio tests or approximations of them; see [8] for the relation of partial correlation test and *F* test, and [1] for the connections of the *G* test to log-linear models and likelihood-ratio tests.

Likelihood-ratio tests, or asymptotically equivalent approximations thereof such as score tests or Wald tests, can be used to compare the goodness-of-fit of nested statistical models. Examples of statistical models are linear regression, binary logistic regression, multinomial regression, and ordinal regression. Two models are called nested, if one model is a special case of the other. Let $M_0$ (reduced model) be a model for *X* using $\mathbf {Z}$, and $M_1$ (full model) be a model for *X* using $Y \cup \mathbf {Z}$. $M_0$ is nested within $M_1$, as $M_1$ can be transformed into $M_0$ by simply setting the coefficients of *Y* to zero. We proceed with a brief description of the likelihood-ratio test; implementation details are considered in Sect. 2.3. Let $\textsc {LL}(M)$ be the log-likelihood of a model *M*, and let $\textsc {Par}(M)$ be the number of parameters in *M*. The test statistic *T* of a nested likelihood-ratio test between $M_0$ and $M_1$ equals $T = 2 \cdot (\textsc {LL}(M_1) - \textsc {LL}(M_0))$ and follows asymptotically a $\chi ^2$ distribution with $\textsc {Par}(M_1) - \textsc {Par}(M_0)$ degrees of freedom [42]. It is important to note that this result assumes that the larger hypothesis $M_1$ is correctly specified, that is, that its assumptions are met (such as functional form and distribution assumption) and that all necessary variables are included. In case of model misspecification, the likelihood-ratio test statistic follows a different distribution [12] and should be handled appropriately [40, 41]. This topic is out of the scope of the current paper and will not be further considered hereafter.

Note that if the models $M_0$ and $M_1$ fit the data equally well and thus are equivalent, it implies that *X* and *Y* are conditionally independent given $\mathbf {Z}$ (assuming again, correct model specification), as *Y* does not provide any additional information for *X* once $\mathbf {Z}$ is given. We will use this property to show how to implement conditional independence tests for mixed variable types in Sect. 4.

### Implementing likelihood-ratio tests with mixed data

Without loss of generality, we assume hereafter that *Y* is the outcome variable, and likelihood-ratio tests are performed using regressions on *Y*. In order to fit a regression model *M* for variable *Y* using mixed continuous, nominal, and ordinal variables, the nominal and ordinal variables have to be first transformed appropriately. Let *X* be a categorical (nominal or ordinal) variable, taking $d_X$ distinct values. *X* can be used in *M* by transforming *X* into $d_X - 1$ dummy binary variables (also called indicator variables). Note that $d_X - 1$ variables are used instead of one for each value of *X*, as the excluded one can be determined given the others. The degrees of freedom of variable *X* is denoted as $\textsc {Dof}(X)$ and equals 1 for continuous variables and $d_X - 1$ for categorical variables. Similarly, the degrees of freedom for a set of variables $\mathbf {Z}$ is defined as $\textsc {Dof}(\mathbf {Z}) = \sum _i \textsc {Dof}(Z_i)$. We note that we only consider linear models with intercept terms and no interaction terms, but everything stated can be directly applied to models with interaction or nonlinear terms.

#### Linear regression

Linear regression models can be used if *Y* is continuous. The number of parameters of the reduced model $M_0$ equals $\textsc {Par}(M_0) = \textsc {Dof}(\mathbf {Z}) + 1$, whereas for $M_1$, $\textsc {Par}(M_1) = \textsc {Dof}(\mathbf {Z}) + \textsc {Dof}(X) + 1$. Typically, *F* tests are used for linear regression. The *F* statistic is computed as

$$\begin{aligned} F = \frac{(RSS_0 - RSS_1) (n-\textsc {Par}(M_1))}{RSS_1 (\textsc {Par}(M_1) - \textsc {Par}(M_0))}, \end{aligned}$$

where $RSS_0$ and $RSS_1$ are the residual sum of squares of models $M_0$ and $M_1$, respectively, and *n* is the sample size. The *F* statistic, under the null hypothesis (the reduced model is the correct one), follows an F distribution with ($\textsc {Par}(M_1) - \textsc {Par}(M_0), n-\textsc {Par}(M_1)$) degrees of freedom, which is asymptotically equivalent to a $v \chi ^2$ distribution with $v =\textsc {Par}(M_1) - \textsc {Par}(M_0) = \textsc {Dof}(X)$ degrees of freedom. Alternatively, if *X* is also continuous, only the full model is required and a *t* test on the coefficient of *X* can be performed.^1^

#### Logistic regression

In case *Y* is nominal, a binary or multinomial logistic regression model can be used, while for ordinal *Y*, ordinal logistic regression is more appropriate. Typically, ordinal logistic regression makes the proportional odds assumption (also known as ordered logit regression): All levels of the ordinal variable must have the same slope, and the estimated constants are nondecreasing. The proportional odds model that *Y* has a value larger than *j* given a set of predictors $\mathbf {X}$ is

$$\begin{aligned} P(Y > j) = \frac{\exp (a_j + \sum _i \beta _i X_i)}{1 + \exp (a_j + \sum _i \beta _i X_i)} \end{aligned}$$

Notice that the values of $\beta _i$ are the same for each category of *Y* (i.e., the log-odds functions for each class of *Y* are parallel). In practice, the proportional odds assumption does not necessarily hold [2]. Because of that, we consider the generalized ordered logit model [43] hereafter, which does not make the proportional odds assumption. The generalized ordered logit model is

$$\begin{aligned} P(Y > j) = \frac{\exp (a_j + \sum _i \beta _{i,j} X_i)}{1 + \exp (a_j + \sum _i \beta _{i,j} X_i)} \end{aligned}$$

where $\beta _{i,j}$ is the coefficient of the *i*-th variable $X_i$ for the *j*-the category of *Y*. Williams [43] described a simple way to fit this model. This is done by fitting a series of binary logistic regressions, where the categories of *Y* are combined. If there are $M=4$ categories for example, then for $j=1$, category 1 is contrasted with categories 2, 3, and 4; for $j=2$, the contrast is between categories 1 and 2 versus 3 and 4; and for $j=3$, it is categories 1, 2, and 3 versus category 4.

Finally, for both multinomial regression and ordinal regression (using the generalized ordered logit model), the number of parameters is $\textsc {Par}(M_0) = (d_Y - 1) (\textsc {Dof}(\mathbf {Z}) + 1)$ and $\textsc {Par}(M_1) = (d_Y - 1) (\textsc {Dof}(\mathbf {Z}) + \textsc {Dof}(X) + 1)$, and the likelihood-ratio test has $(d_Y - 1) \textsc {Dof}(X)$ degrees of freedom.

#### Limitations

We note that *we implicitly assume that the assumptions of the respective models hold*. For instance, linear regression assumes (among others) independent and Gaussian residuals, homoscedasticity and that the outcome is a linear function of the model variables. The latter also applies to logistic regression models, and specifically that the log-odds ratio is a linear function of the variables. If the model assumptions do not hold, the tests do not follow the same asymptotic distribution, and thus may lead to different results. However, we note that linear regression models are robust to deviations from the assumption of normality of residuals, and to a smaller degree to deviations of the homoscedasticity assumption [26]. The latter could also be handled by using tests based on robust regression.

**Fig. 1:**
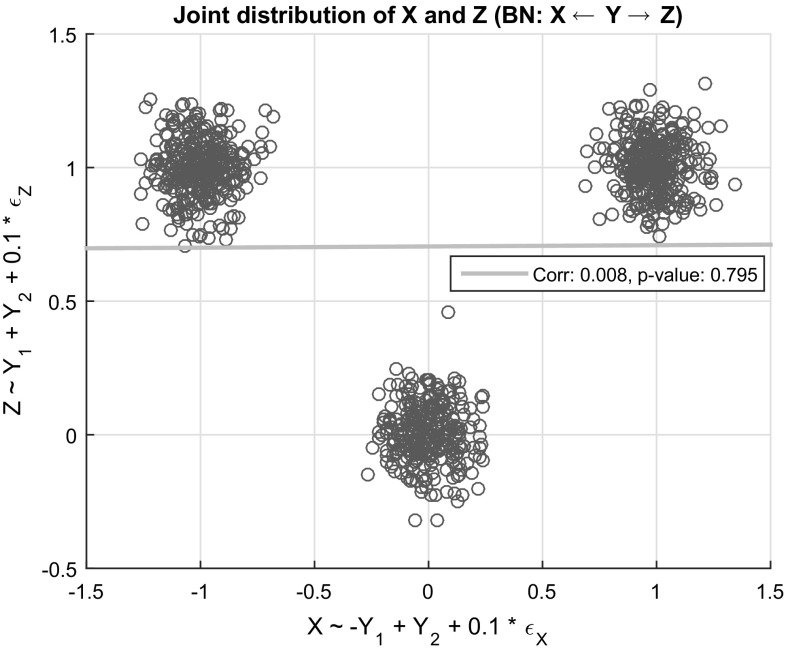
An example where the proposed tests fail to identify the unconditional dependency between *X* and *Z* is shown. The correlation between *X* and *Z* is 0.008, and the *p* value of the test equals 0.795, suggesting independence

Furthermore, we also note that even if the data come from a BN whose functional relations are linear models as the ones considered above, there are cases where tests fail to identify certain dependencies. Consider, for example, a simple network consisting of three variables, *X*, *Y*, and *Z*, where *Y* is nominal with three levels, *X* and *Z* are continuous and *Y* is a parent of *X* and *Z*. Let *Y* be uniformly distributed, $Y_i$ denote the binary variable corresponding to the *i*-th dummy variable of *Y*, $X = -Y_1 + Y_2 + 0.1 \epsilon _X$, and $Z = Y_1 + Y_2 + 0.1 \epsilon _Z$, where $\epsilon ~ N(0,1)$. Thus, the conditional distribution of *X* and *Z* given *Y* is Gaussian, although their marginal distribution is non-Gaussian. An example of the joint distribution of *X* and *Z* with 1000 random samples is shown in Fig. 1. Notice that *Y* induces a nonlinear relation between *X* and *Z*, even though all functions are linear. Therefore, any test based on linear regression models on *X* and *Z* (or equivalently Pearson correlation) will not identify the dependence between them, despite them being unconditionally dependent. One approach to this problem is to use kernel-based tests (or other, nonlinear tests), which would be able to identify such a dependency asymptotically. We note that although indirect dependencies may be missed by the proposed tests, direct dependencies (edges) would still be identified. Thus, algorithms such as the conservative PC algorithm [32] that only rely on the adjacency faithfulness assumption (i.e., two adjacent variables are dependent given any set of variables) could be used in conjunction with those tests, and the results would be correct, although possibly less informative.

## Related work

Mixed data have been considered in the context of Markov network learning; see [44] for a review of such methods. Heckerman et al. [16] were the first to propose a Bayesian method to score BNs with mixed categorical and Gaussian variables. The score is derived under the assumption that continuous variables with discrete parents follow a conditional Gaussian distribution, similar to the graphical models considered by Lauritzen and Wermuth [25]. An important drawback of this approach is that it does not allow discrete variables to have continuous parents, limiting its use in practice. A different approach is followed by Friedman et al. [13, 30], who consider methods of discretization of continuous variables given a specific BNs structure. Such techniques can then be used to search over both, a BN structure and a discretization strategy. Margaritis and Thrun [28] propose a method for testing unconditional independence for continuous variables, which is also directly applicable to ordinal and nominal variables. The method has also been extended to the conditional case, with a single variable in the conditioning set [27]. We are not aware of any extension to the general case that considers larger conditioning sets. Bach and Jordan [4] propose a kernel-based method for graphical model learning with mixed discrete and continuous variables and show how both scores and conditional independence tests can be derived from it. Its main drawbacks are that (a) it has two hyper-parameters, which may be hard to tune and (b) that it is computationally demanding, having a time complexity of $O(n^3)$, where *n* is the sample size, although approximations can be used that scale linearly with sample size.

Cui et al. [10] suggested a copula-based method for performing conditional independence tests with mixed continuous and ordinal variables. The idea is to estimate the correlation matrix of all variables in the latent space (containing latent variables which are mapped to the observed variables), which can then be directly used to compute partial correlations and perform independence tests. To this end, they employ Hoff’s Gibbs sampling scheme for generating covariance matrices using copula [17]. The main disadvantage is that the correlation matrix is estimated using Gibbs sampling and thus may be computationally demanding and hard to estimate accurately. Karra and Mili [23] build upon the work of [11] and propose hybrid copula BNs, which can model both discrete and continuous variables, as well as a method for scoring such networks.

Recently, [34] proposed to use likelihood-ratio tests based on linear and logistic regression models to derive conditional independence tests for mixed continuous and nominal variables. They suggest to use linear regression instead of logistic regression whenever applicable, as it is more accurate. This work is most closely related to our approach. The main differences are: (a) They only consider continuous and nominal variables, whereas our proposed approach is more general and is able to deal with other variable types such as ordinal variables and (b) they do not address the asymmetry between both directional tests, while we propose and evaluate methods that handle it.

## Symmetric conditional independence tests for mixed data

We consider conditional independence tests based on nested likelihood-ratio tests, using linear, logistic, multinomial, and ordinal regression to handle continuous, binary, nominal, and ordinal variables, respectively. For all cases, we only consider models with linear terms, without any interactions, although this is not a limitation of the proposed approach and additional terms can be included.

Let $H_0$: $P(X,Y|\mathbf {Z})$ = $P(X|\mathbf {Z}) \cdot P(Y|\mathbf {Z})$ (X and Y are conditionally independent given $\mathbf {Z}$) be the null hypothesis of conditional independence. Since we do not have a direct way to test this hypothesis, we consider the null hypotheses $H_1$: $P(X|Y,\mathbf {Z}) = P(X|\mathbf {Z})$ and $H_2$: $P(Y|X,\mathbf {Z}) = P(Y|\mathbf {Z})$. $H_1$ can be tested using a nested likelihood-ratio test by regressing on *X*, while $H_2$ can be tested by flipping *X* and *Y* and regressing on *Y*. For instance, if *X* is continuous and *Y* is nominal, one can either fit two linear regression models for *X* to test $H_1$, one using $Y \cup \mathbf {Z}$ (full model) and one using only *Y* (reduced model) and perform an *F* test, or to fit two multinomial logistic regression models for *Y* in a similar fashion to test $H_2$ and perform a likelihood-ratio test. Ideally, both tests should give identical results and thus be symmetric.

There are special cases, such as when *X* and *Y* are continuous and linear regression models are used, where symmetry holds. Unfortunately, this does not necessarily hold in the general case. To the best of our knowledge, it is not known under which conditions such tests are symmetric. Empirical evidence (see Sect. 5) suggests that tests using the aforementioned models give the same results asymptotically (this was also mentioned in [34]). Therefore, given sufficiently many samples, any one of the two tests can be used. For small sample settings, however, the test results often differ, which motivated us to consider methods for deriving symmetric tests.

### Symmetric tests by combining dependent *p* values

One approach is to perform both tests and to combine them appropriately. Let $p_1$ and $p_2$ be the *p* values of the tests for $H_1$ and $H_2$, respectively. As both hypothesis tests essentially test the same hypothesis, one can expect the *p* values to be positively dependent. We use a method presented in [5] for combining dependent *p* values (which we call **MM** hereafter), an extension of a previous method [35]. The resulting *p* value $p_{mm}$ is computed as

1 $$\begin{aligned} p_{mm} = \min \left\{ 2 \min (p_1,p_2), \max (p_1, p_2) \right\} . \end{aligned}$$

This *p* value can be used to assess whether at least one of the two asymmetric null hypotheses can be rejected. Moreover, it can be demonstrated that $p_{mm}$ is theoretically correct even in the presence of specific types of correlations among the two *p* values, as in the case of one-sided *p* values based on Gaussian test statistics that are positively correlated [5]; whether this also holds for combining *p* values stemming from tests considered here is not clear and needs further investigation, but it is nevertheless a useful heuristic. In addition to that, we considered two simple approaches, by taking the minimum or the maximum between the two *p* values

2 $$\begin{aligned} p_{min}= & {} \min (p_1, p_2) \ \ \text {or} \end{aligned}$$

3 $$\begin{aligned} p_{max}= & {} \max (p_1, p_2) \end{aligned}$$

The latter is identical to testing whether both hypotheses can be rejected and is an instance of the method by Benjamini and Heller [5] for combining dependent *p* values. Although taking the minimum *p* value should be avoided for independent *p* values, as it does not account for multiple testing, it may be a reasonable choice if the *p* values have a high positive correlation.

There has been theoretical work for deriving the true distribution of the sum or the ratio of the two test statistics, assuming their correlation is known [19, 20]. A general, permutation-based method for estimating the correlation between test statistics has been proposed by Hongying Dai and Cui [18]. This is computationally expensive, as it requires fitting a large number of models, which is prohibitive for learning graphical models. In anecdotal experiments, we found that this method and the ones considered above produce similar results, and thus it was not further considered.

### A strategy for prioritizing asymmetric tests

A different approach for deriving symmetric tests is to use a strategy to prioritize tests and to only perform one of the two tests. This is especially attractive due to its lower computational cost, compared to the previously described approach. Sedgewick et al. [34] compared tests based on linear regression and multinomial logistic regression and found that linear regression is generally more accurate. This can be explained by the fact that the full linear regression model has fewer parameters to fit than the full multinomial regression model (unless the variable is binary) and thus can be estimated more accurately given the same amount of samples. Let *X* be a continuous variable, and *Y* be a categorical (nominal or ordinal) variable taking $d_Y$ values. The number of parameters required by a full linear regression model for *X* using *Y* and $\mathbf {Z}$ equals $\textsc {Dof}(\mathbf {Z}) + (d_Y - 1) + 1$ (see Sect. 2.3). The logistic regression model for *Y* on the other hand requires $(d_Y - 1) \cdot (\textsc {Dof}(\mathbf {Z}) + 1 + 1)$ parameters. Thus, unless *Y* is binary and $d_Y = 2$, the logistic regression model always contains more parameters. Everything stated above also holds for the case of unconstrained generalized ordinal regression models. Using this fact, and the observation made by Sedgewick et al. [34], we propose to prioritize tests as follows.

$$\mathbf{Priority: }\; \text {Continuous}> \text {Nominal} > \text {Ordinal}$$

In case of two nominal or ordinal variables, the variable with the fewer values is regressed on, while in case of ties, an arbitrary variable is picked. Note that if the latter holds, the proposed strategy is not always symmetric; we plan to address this case in future work. Recall that if both *X* and *Y* are continuous, the tests are symmetric and thus any one of them can be used. In anecdotal experiments, we observed that ordinal regression models, especially the ones considered here, are typically harder to fit than multinomial logistic models, which is the reason why we prioritize nominal over ordinal variables. Hereafter, we will refer to this approach as the **Fast** approach.

Finally, we note that the problem of asymmetry has been addressed before in different contexts. The MMHC algorithm [39] for BN learning performs feature selection for each variable using the MMPC algorithm to identify a set of candidate parents and children (neighbors), which may result in cases where a variable *X* is a neighbor of another variable *Y* but the opposite does not hold. If this is the case, MMPC corrects the asymmetry by removing variable *X* from the set of neighbors of *Y*. Similar, in the context of Markov networks, Meinshausen and Bühlmann [29] consider adding an edge between two variables if their neighbor sets contain each other (logical conjunction) or if at least one of the neighbor sets contains the other (logical disjunction), where neighbor sets are inferred independently for each node. The authors state that both approaches perform similarly and are asymptotically identical. Both methods use asymmetric tests to identify the neighbors of each node and then perform a symmetry correction. This approach is similar, although not exactly the same, as taking the minimum (logical disjunction) or maximum (logical conjunction) *p* value. Both are valid strategies, and should perform similarly (at least for large sample sizes) to the proposed ones. However, the proposed strategies are more general, thus applicable with any method that uses conditional independence tests. In Sect. 5, we also see that the MM typically performs better than strategies based on taking the minimum or maximum *p* value.

### Limitations

For certain variable types, such as longitudinal and censored time-to-event data, it is not always possible to perform both tests. Unless both *X* and *Y* are of the same type (e.g., both longitudinal or censored time-to-event), it is not clear how to regress on the nontime-related variable. For example, if *X* is a censored time-to-event variable (that is, a binary variable indicating whether an event occurred, as well a continuous variable with the time of the event), and *Y* is a continuous variable, it is straightforward to regress *Y* on *X* using methods such as Cox regression to perform a likelihood-ratio test, while the opposite is harder to handle. We plan to investigate such variable types in the future.

## Simulation studies

We conducted experiments on simulated data to investigate the properties of mixed tests based on regression models, and to evaluate the proposed symmetric tests. Afterward, we compare the MM and Fast symmetric tests to a copula-based approach (called **Copula** hereafter) for mixed continuous and ordinal variables [10] in the context of BN learning. The methods were compared on synthetic BNs with continuous and ordinal variables.

### Data generation

We proceed with a description of the data generation procedure used throughout the experiments. We will describe the general case for data generation given a BN structure *G* and the type of each variable. Let *X* be a variable in *G*, and *Pa*(*X*) be the parents of *X* in *G*. For the moment, we will only consider continuous and ordinal variables; ordinal variables will be treated separately afterward. In all experiments, ordinal variables take up to four values.

In case *Pa*(*X*) is empty, *X* is sampled from the standard normal distribution if it is continuous, and is uniformly distributed in case it is binary/nominal. If *Pa*(*X*) is not empty, then $X = f(Pa(X)) = f(b_0 + \sum _i b_i Pa_i(X) + \epsilon _X)$, which is a linear or generalized linear function depending on the type of *X*. Although not shown above, as before all nominal variables are transformed into dummy variables, and thus a coefficient is assigned to each dummy variable. The following procedure is used to generate data for *X*.

1. Generate samples for each variable in *Pa*(*X*) recursively, until samples for each variable are available
2. Sample the coefficients *b* of *f*(*Pa*(*X*)) uniformly at random from $[-1,-0.1] \cup [0.1,1]$
3. Generate $$\epsilon _X \sim N(0,1)$$
4. Compute *X* using *f*(*Pa*(*X*))

In order to generate ordinal variables, we first generated a continuous variable as described above and then discretized it into 2–4 categories appropriately (without damaging the ordered property). Each category contains at least 15% of the observations, while the remaining ones are randomly allocated to all categories. This is identical to having a latent continuous variable (the one generated), but observing a discretized proxy of it. Note that, as the discretization is random, any normality of the input continuous variable is not preserved. Finally, ordinal variables in the parent sets are not treated as nominal variables, but simply as continuous ones and thus only one coefficient is used for them for the purpose of data generation.

### Investigating the properties of mixed tests

We considered five combinations of variable types and corresponding regression models: (a) linear-binary (L-B), (b) linear -multinomial (L-M), (c) linear-ordinal (L-O), (d) binary-ordinal (B-O), and (e) multinomial-ordinal (M-O). For each case, we considered the following simple BN models: (a) X   Y (unconditional independence), (b) $X\rightarrow Y$ and $X \leftarrow Y$ (unconditional dependence), (c) $X\rightarrow Z \leftarrow Y$ (conditional dependence of *X* and *Y* given *Z*), also known as collider [37], and (d) $X\leftarrow Z \rightarrow Y$ (conditional independence of *X* and *Y* given *Z*). In all cases, *Z* is continuous. We used the previously described procedure to generate data for those networks. The sample size varied in (20, 50, 100, 200, 500, 1000), and each experiment was performed 1000 times, except for case (b), which was performed 1000 times for each direction.

**Fig. 2:**
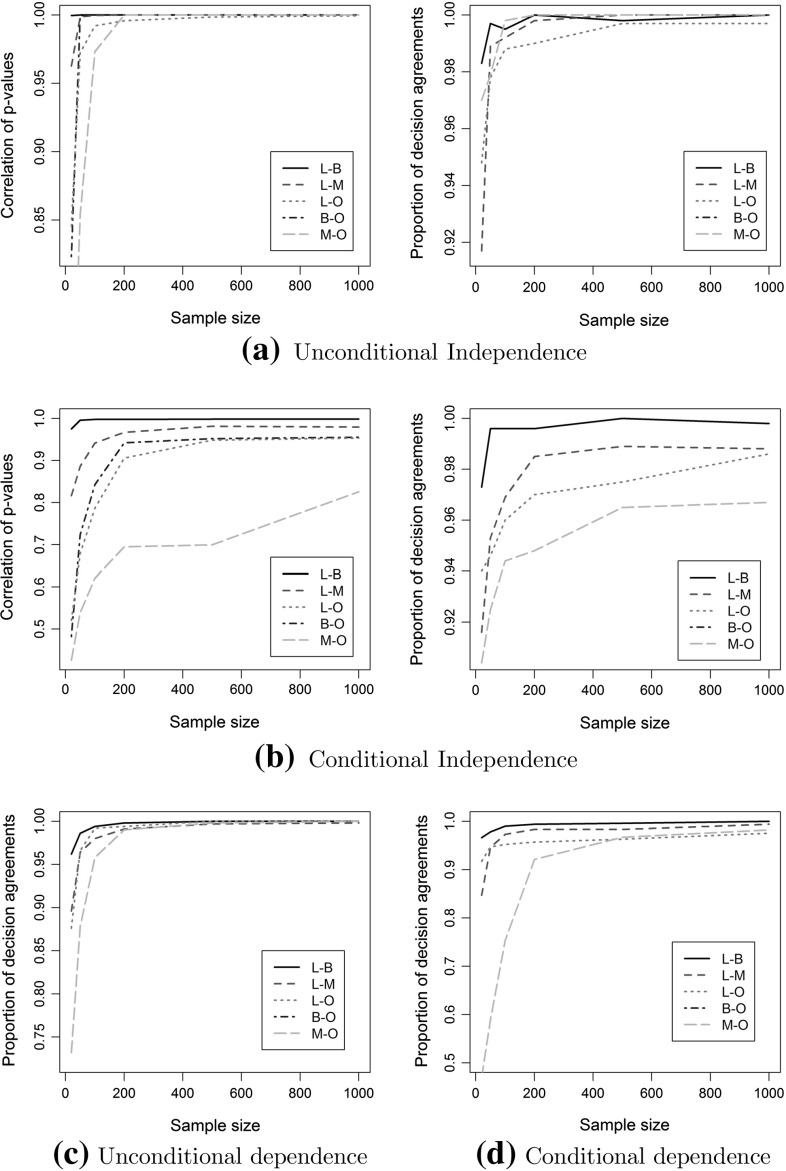
The correlation of the two *p* values and the proportion of decision agreements at the 5% significance level are shown for different pairs of regression models. The correlation of *p* values for (un)conditional independence increases with sample size, reaching almost perfect positive correlation in most cases. In terms of decision agreements, an agreement of over 90% is reached in all cases even with 200 samples. **a** Unconditional independence, **b** conditional independence, **c** unconditional dependence, and **d** conditional dependence

**Fig. 3:**
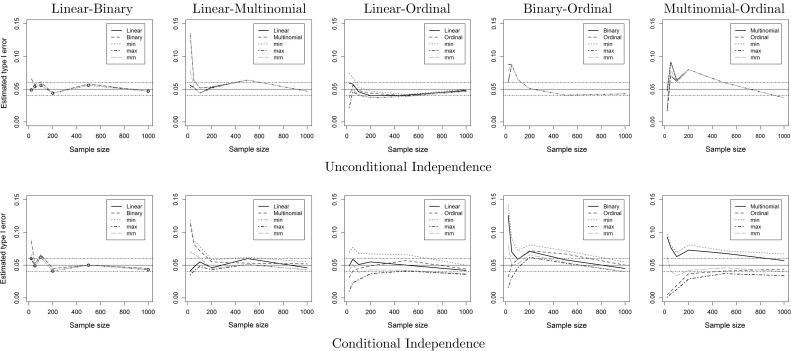
Estimated type I error on the (un)conditional independence cases for each pair of regression models, and three methods for combining dependent *p* values. The solid horizontal line is at the 5% level, and the two dashed lines at 4 and 6% levels. Whenever linear regression models are involved, the MM method and the linear test perform similarly. For the conditional case of binary-ordinal and multinomial-ordinal pairs, the MM method outperforms all methods

**Fig. 4:**
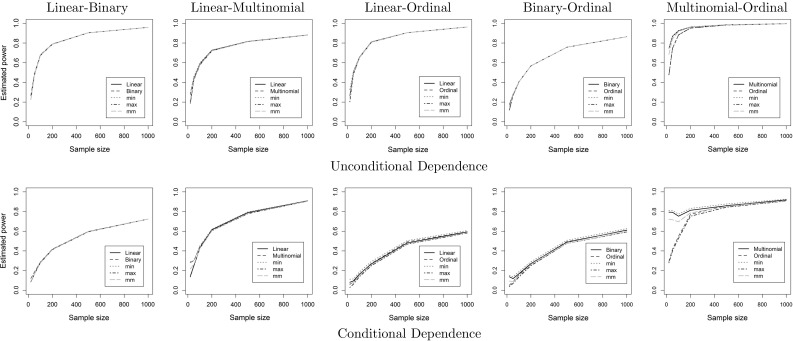
Estimated power on the (un)conditional dependence cases for each pair of regression models, and three methods for combining dependent *p* values. In most cases, all methods perform very similar. For the multinomial-ordinal case, ordinal regression breaks down for small samples, and MM is slightly behind the rest. This is expected, as the other methods also have a larger type I error

Figure 2 shows the correlation and decision agreements (reject or not the null hypothesis) at the 5% significance level (similar results hold true for the 0.1, 1, and 10% significance levels) between all five pairs of regression models. For the unconditional dependence case, in which both directional models were considered, we repeated the experiment twice and report averages over both cases. We did not consider the correlation of *p* values in dependent cases, as one is typically interested to have low enough *p* values to reject the null hypothesis. Overall, the correlation between both tests is very high and tends to one with increasing sample size. An exception is the multinomial-ordinal (M-O) conditional independence case, whose correlation is noticeably smaller than the rest. This can be explained by the fact that this test is the hardest one, as either test uses models with 15 parameters to be fit, requiring more samples. The proportion of decision agreements is very high for all pairs, reaching over 90% even with 200 samples. This is very encouraging, as this is the most important factor for causal discovery methods.

**Table 1:** Precision and recall for the skeleton estimation

Method	50 variables			100 variables		
	$n=200$	$n=500$	$n=1000$	$n=200$	$n=500$	$n=1000$
*Skeleton precision*						
3 neighbors						
MM	0.783	0.981	0.988	0.949	0.971	0.974
Fast	0.708*	0.971*	0.979*	0.936*	0.952*	0.951*
Copula	*0*.*898*	0.942*	0.975	0.884*	0.896	0.914*
5 neighbors						
MM	0.989	0.992	0.993	0.988	0.992	0.989
Fast	0.986	0.990	0.992	0.984	0.985*	0.985*
Copula	0.980*	0.971*	0.951*	0.987	0.961*	0.950*
*Skeleton recall*						
3 neighbors						
MM	0.172	0.704	0.808	0.536	0.707*	0.794
Fast	0.155*	0.639*	0.711*	0.507*	0.643*	0.684*
Copula	0.152*	0.675	0.796	0.402*	0.669*	0.793
5 neighbors						
MM	0.445	0.617	0.717	0.460	0.624	0.725
Fast	0.436*	0.575*	0.649*	0.457	0.582*	0.660*
Copula	0.374*	0.600*	0.700	0.341*	0.595*	0.725

An asterisk (*) indicates that the precision or recall of the Fast or Copula approach is statistically significantly lower than that of MM at the 1% significance level. The italic font indicates that the precision of the Copula approach is statistically significantly higher than that of MM at 1% significance level

Figures 3 and 4 show the estimated type I error and power of all methods. In the unconditional cases, as well as in most conditional cases, all methods perform similarly. Whenever linear models are involved, the asymmetric linear test and the symmetric MM method outperform the rest, which can be seen mostly in the type I error on the conditional independence case. For the conditional case of binary-ordinal and multinomial-ordinal pairs, the MM method offers the best trade-off between type I error, as it very close to 5%, and power, being only slightly worse than some competitors for small samples. Asymmetric tests based on ordinal regression break down in the conditional cases for small sample sizes, and symmetric methods like MM should be preferred.

### Evaluation on Bayesian network learning

As shown in the previous section, the best performing method is the MM method, while the proposed asymmetric approach seems to be promising if continuous variables are involved. In this section, we use those methods for BN learning. We compare them to a recent method by Cui et al. [10], which is applicable to continuous, binary, and ordinal variables. As a BN learning algorithm, we used the order-independent PC algorithm [21], as implemented in the R package *pcalg* [22]. The significance level was set to 0.01 for all experiments. For the Copula method, [10] used 20 burn-in samples, and 80 samples to estimate the correlation matrix using Gibbs sampling. We increased these numbers to further improve its accuracy. Specifically, we used 2*p* burn-in samples and 4*p* samples to estimate the correlation matrix, where *p* is the number of variables in the data.

We generated BNs with 50 and 100 variables, and with an average degree of 3 and 5. For each case, we generated 50 random BNs and sampled 200, 500, and 1000 training instances. In total, this amounts to 600 datasets. Each variable has a 50% probability of being continuous or ordinal, and ordinal variables take 2–4 values with equal probability. The sampling of the network parameters and the data generation were performed as described above.

**Table 2:** Precision and recall for the estimation of the orientations

Method	50 variables			100 variables		
	$n=200$	$n=500$	$n=1000$	$n=200$	$n=500$	$n=1000$
*Orientation precision*						
3 neighbors						
MM	0.686	0.979	0.988	0.943	0.965	0.974
Fast	0.608*	0.969*	0.978*	0.928*	0.942*	0.948*
Copula	*0*.*812*	0.940*	0.928*	*0*.*976*	0.932*	0.913*
5 neighbors						
MM	0.987	0.992	0.993	0.986	0.992	0.989
Fast	0.984	0.989	0.992	0.982	0.985*	0.984*
Copula	0.975*	0.970*	0.950*	0.984	0.959*	0.949*
*Orientation recall*						
3 neighbors						
MM	0.118	0.692	0.806	0.504	0.668	0.790
Fast	0.108*	0.621*	0.698*	0.476*	0.600*	0.669*
Copula	0.092*	0.666	0.793	0.342*	0.625*	0.791
5 neighbors						
MM	0.413	0.606	0.711	0.430	0.613	0.719
Fast	0.406*	0.561*	0.638*	0.428	0.569*	0.649*
Copula	0.327*	0.591	0.696	0.289*	0.583*	0.722

An asterisk (*) indicates that the precision or recall of the Fast or Copula approach is statistically significantly lower than that of MM at the 1% significance level. The italic font indicates that the precision of the Copula approach is statistically significantly higher than that of MM at 1% significance level

**Table 3:** Structural Hamming distance (lower is better)

Method	50 variables			100 variables		
	$n=200$	$n=500$	$n=1000$	$n=200$	$n=500$	$n=1000$
*Structural Hamming distance*						
3 neighbors						
MM	71.48	34.40	25.64	97.62	69.94	55.12
Fast	73.18*	38.66*	33.76*	101.04*	80.46*	74.28*
Copula	70.96	37.12*	30.30*	115.66*	76.88*	62.00*
5 neighbors						
MM	81.46	57.28	44.54	158.60	112.35	87.10
Fast	82.12	62.62*	53.56*	158.50	123.55*	105.30*
Copula	91.60*	60.42*	49.84*	191.40*	124.95*	93.15*

An asterisk (*) indicates that the SHD of the Fast or Copula approach is statistically significantly higher than that of MM at the 1% significance level

To evaluate the performance of the different methods, we computed the structural Hamming distance (SHD) [39], as well as the precision and recall of the network structure and orientations. Naturally, all metrics were computed on the estimated PDAG and the true PDAG. Precision and recall are proportions; hence, in order to compare their values, we used the *t* test applied on $\log {\frac{a}{b}}$, where *a* is the precision (recall) of the MM method, and *b* is the corresponding precision (recall) of the competing methods.^2^ As for the SHD, we took the differences between the MM method and the rest. Since the values of the SHD are discrete, we used the Skellam distribution [36] and tested (via a likelihood-ratio test) whether its two parameters are equal, implying that the compared values are equal. A *t* test could be applied here as well, but in order to be more exact, we used a test (or distribution) designed for discrete data.

The results are summarized in Tables 1, 2, and 3. Each table contains average values over 50 random BNs. Overall, the proposed MM approach statistically significantly outperforms the other methods across all computed performance metrics. The Copula method outperforms MM in terms of both prediction metrics only in the 50-variable case with average degree 3 and 200 samples, while the Fast approach is always inferior to MM and is often comparable to Copula. Furthermore, both MM and Fast improve across all metrics with increasing sample size. Copula, however, does not always do so, and precision often declines with increasing sample size (e.g., see cases with 50 variable networks).

## Conclusions

In this paper, a general method for conditional independence testing on mixed data is proposed, such as mixtures of continuous, nominal, and ordinal variables, using likelihood-ratio tests based on regression models. Likelihood-ratio tests are not necessarily symmetric, and different approaches to derive symmetric tests are considered. In simulated experiments, it is shown that the likelihood-ratio tests considered in this paper are asymptotically symmetric. Furthermore, the proposed symmetric MM test is shown to significantly outperform competing methods in BN learning tasks. R codes to learn BNs with mixed data are available at the R package *MXM* [24].

## Footnotes

1 In this case, the *t* test (Wald test) is equivalent to the *F* test (likelihood-ratio test).

2 The reason of this is that, these measure being proportions, the absolute difference does not reflect the same information as the ratio which is more meaningful. The difference, for example, between 0.2 and 0.1 is the same as that of 0.8 and 0.7, but their ratio is clearly not the same.
